# Dysregulation of the miR-148a–GLUT1 axis promotes the progression and chemoresistance of human intrahepatic cholangiocarcinoma

**DOI:** 10.1038/s41389-020-0207-2

**Published:** 2020-02-13

**Authors:** Pei Tiemin, Xiao Peng, lang Qingfu, Wang Yan, Xue Junlin, He Zhefeng, Zhao Ming, Liang Desen, Meng Qinghui

**Affiliations:** Department of General Surgery, Key Laboratory of Hepatosplenic Surgery, Ministry of Education, The First Affiliated Hospital of Harbin Medical University, Harbin, China

**Keywords:** Metastasis, Oncogenes

## Abstract

Intrahepatic cholangiocarcinoma (iCCA) is a highly fatal malignant cancer worldwide. Elucidating the underlying molecular mechanism of iCCA progression is critical for the identification of new therapeutic targets. The present study explored the role of the miR-148a–GLUT1 axis in the progression of iCCA. The expression of GLUT1 was detected by using immunohistochemistry, western blot assays, and real-time polymerase chain reaction. The effects of GLUT1 on cell proliferation, invasion, and chemoresistance were investigated both in vitro and in vivo. A luciferase reporter assay was used to explore the effect of miR-148a on GLUT1 expression. GLUT1 was overexpressed in iCCA tissues. GLUT1 overexpression was associated with shorter overall and disease-free survival. Knockdown of GLUT1 reduced, while overexpression of GLUT1 promoted, the proliferation, motility, and invasiveness of iCCA cells in vitro and in vivo. Silencing GLUT1 significantly sensitized iCCA cells to gemcitabine in vitro and in vivo. GLUT1 was directly regulated by miR-148a, whose downregulation was associated with the proliferation, migration, and invasion of iCCA cells. WZB117, a GLUT1 inhibitor, inhibited tumor growth in an iCCA patient-derived xenograft model. These results indicate that downregulation of miR-148a levels results in GLUT1 overexpression in iCCA, leading to iCCA progression and gemcitabine resistance.

## Introduction

Intrahepatic cholangiocarcinoma (iCCA) is the second most common primary hepatic malignancy^1^. In addition to being one of the most aggressive forms of cancer, the incidence and mortality of iCCA have been rapidly increasing worldwide over the past decade^2^. Surgical resection remains the most efficient treatment for patients with iCCA, but the tumors are often quite advanced at the time of diagnosis and surgical resection is not usually possible^3^. Even worse, no effective chemotherapies or molecular target therapies are available for iCCA, which has been mainly attributed to a poor understanding of the molecular mechanisms of this malignancy^4–6^. Therefore, elucidating the underlying mechanisms of iCCA progression is crucial to improve patient survival.

In 1924, Otto Warburg described glucose metabolism in cancer, which is currently referred to as the “Warburg effect”^4^ and has recently been indicated as a general hallmark of cancer. Cancer cells aberrantly express some specific proteins involved in glucose metabolism, such as glucose transporter 1 (GLUT1), pyruvate kinase M2 (PKM2), and hexokinase 2 (HK2)^5^. GLUT1 is a key rate-limiting factor for the transport and metabolism of glucose in cancer cells. The overexpression of GLUT1 has also been reported in cancer cells, and high levels of GLUT1 have been associated with poor outcomes in cancer patients^6–10^. Several oncogenic transcription factors, such as c-Myc, have been shown to directly regulate GLUT1 mRNA expression in human cancers^11^. Moreover, the selective acquisition of KRAS or BRAF mutations in response to glucose deprivation has been shown to upregulate GLUT1 expression^12^. In addition, GLUT1 expression is regulated by tumor microenvironmental effectors. For example, hypoxia induces GLUT1 expression via the transcription factor hypoxia-inducible factor-1 (HIF-1)^13^. To date, only a few studies have addressed the expression of GLUT1 in iCCA^14^, and the clinical significance and role of GLUT1 in iCCA remain unknown.

MicroRNAs (miRNAs) are small single-stranded noncoding RNAs (21–23 nucleotides long) that are encoded in the genomes of plants, invertebrates, and vertebrates. MicroRNAs exert their functions by directly binding to the 3′-untranslated region (3′-UTR) of mRNAs and subsequently negatively regulating gene expression posttranscriptionally^15^. Increasing evidence has demonstrated that aberrant miRNA levels are involved in various cancers, including iCCA, and play critical roles in tumorigenesis and metastasis^16,17^. The levels of miRNA-191 are upregulated in iCCA and promote the malignant phenotype of iCCA cells by the targeted modulation of the TET1-p53 pathway^18^. In iCCA, UHRF1 is targeted by miR-124-3p and promotes cell proliferation^19^. MiR-148a has recently been associated with several human cancers, with its role appearing to be tissue-specific. The downregulated expression of miR-148a can be detected in various cancers, including gastric, colorectal, pancreatic, liver, esophageal, breast, non-small cell lung, and urogenital system cancers. However, the upregulated expression of miR-148a can also be observed in glioma and osteosarcoma^20^. Nonetheless, the role of miR-148a in iCCA remains unknown. In this study, we evaluated the role of the miR-148a–GLUT1 axis in the progression of iCCA.

## Results

### GLUT1 is upregulated in human iCCA and correlated with poor clinical outcomes

**Fig. 1:**
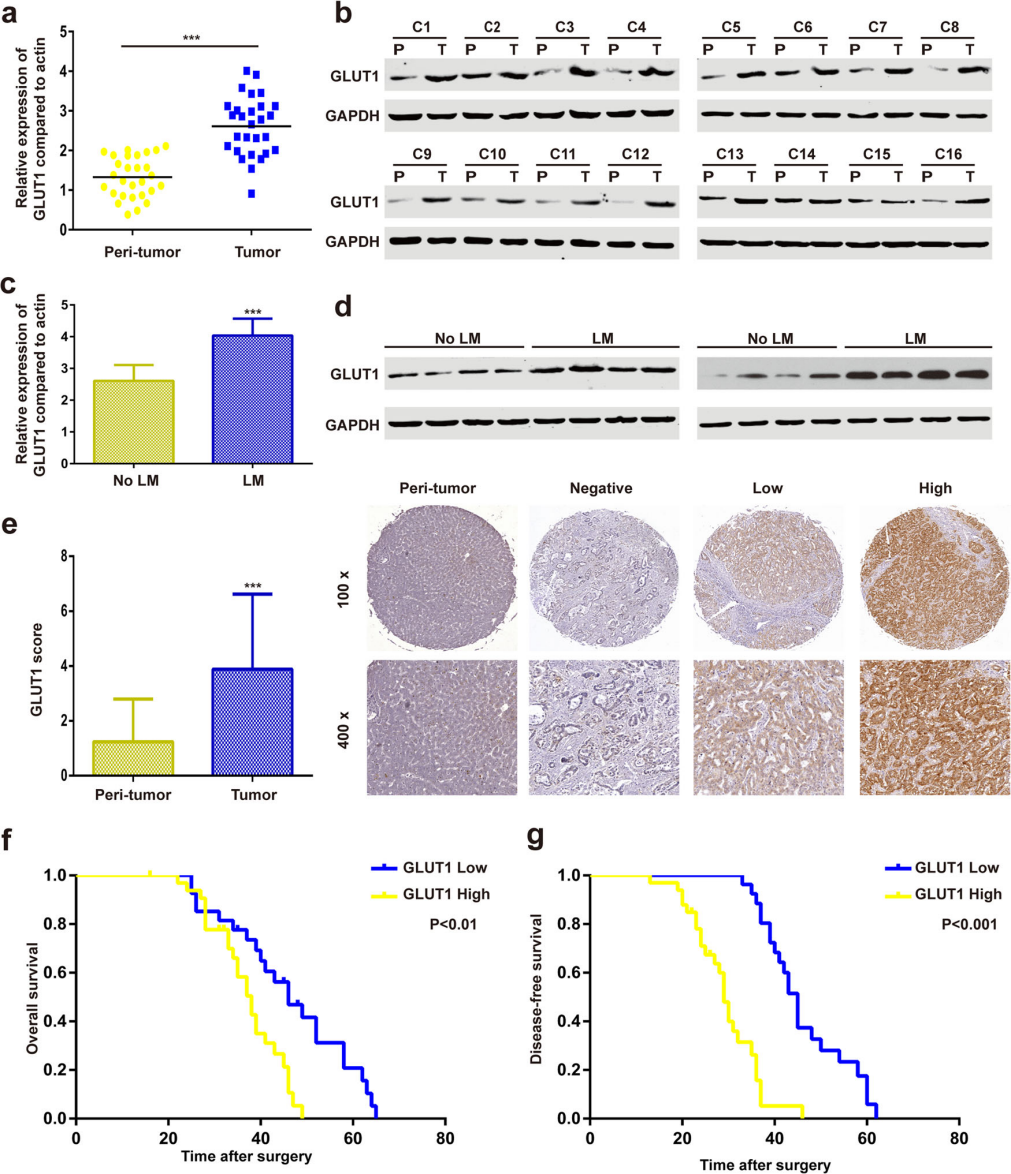
GLUT1 is upregulated in human iCCA and correlated with poor clinical outcomes. **a** GLUT1 mRNA levels were analyzed in 60 iCCA and paracancerous specimens using real-time quantitative reverse transcription-polymerase chain reaction (qRT-PCR). **b** Western blot analyses of GLUT1 protein expression in iCCA tissues and adjacent normal tissues. **c** and **d** GLUT1 expression levels in iCCA with and without lymph node metastasis were evaluated by qRT-PCR and western blot assays. **e** Immunohistochemical (IHC) staining of GLUT1 protein expression in iCCA tissues and adjacent normal tissues. **f** and **g** Kaplan–Meier survival analyses were conducted to assess the influence of GLUT1 on overall or disease-free survival. Data are means ± SD of three independent experiments. ***P < 0.001.

Real-time PCR (RT-PCR) of 28 paired iCCA samples and matched adjacent non-tumor liver tissues showed that the mRNA expression of GLUT1 was upregulated in iCCA tissues (Fig. 1a). Western blot assays of 16 paired iCCA samples and matched adjacent non-tumor liver tissues produced similar results (Fig. 1b).

Compared to other tumor types, lymphatic metastasis is more prevalent in iCCA and is an important predictive factor for poor prognosis^21^. Therefore, we evaluated another eight independent iCCA samples, with or without lymphatic metastasis, and found that GLUT1 mRNA and protein expression were higher in iCCA with lymphatic metastasis than in those without lymphatic metastasis (Fig. 1c, d). Immunohistochemistry (IHC) results from the 60 paired iCCA samples and matched adjacent non-tumor liver tissues showed that the GLUT1 staining score of the iCCA tissues was higher than that of the adjacent non-tumor tissues (Fig. 1e). According to the IHC results, the 60 patients with iCCA were divided into either the high-expression (33 patients) or low-expression group (27 patients). Twenty-eight patients in the high-expression group had lymphatic metastasis, whereas nine patients in the low-expression group had lymphatic metastasis. High GLUT1 expression had shorter overall and disease-free survival than those with low GLUT1 expression (Fig. 1f, g).

### Inhibition of GLUT1 suppresses iCCA proliferation and cell cycle progression in vitro

To evaluate the impact of GLUT1 expression on iCCA progression, two cholangiocarcinoma cell lines, HuCCT1 and RBE, were stably transfected with lentiviral vector encoding short hairpin GLUT1 (sh GLUT1) or lentiviral vector encoding wild-type GLUT1. GLUT1 overexpression and downregulation efficiency were confirmed using RT-PCR and western blot assays (Supplementary Fig. 1). GLUT1 was strongly downregulated by lenti-shRNA1 (KD-1) and moderately downregulated by KD-2 and KD-3 compared to the control shRNA. Therefore, KD-1 was chosen for further experiments.

Growth curve assays showed that GLUT1 overexpression enhanced the proliferation of RBE cells, whereas GLUT1 knockdown suppressed the cell growth of HuCCT1 cells (Supplementary Fig. 2a). Cell cycle analysis showed that the inhibition of GLUT1 significantly decreased G1-to-S phase cell cycle progression in HuCCT1 cells, whereas the overexpression of GLUT1 increased G1-to-S phase cell cycle progression in RBE cells (Supplementary Fig. 2b). Colony formation assays showed that the overexpression of GLUT1 increased the number and colonies formed by RBE cells, whereas the inhibition of GLUT1 reduced the number of colonies formed by HuCCT1 cells (Supplementary Fig. 2c).

Several cell cycle regulators, including cyclin D1, p27, and p21, were investigated. GLUT1 knockdown markedly decreased the levels of cyclin D1, while GLUT1 overexpression increased the abundance of cyclin D1 (Supplementary Fig. 2d). Silencing or overexpressing GLUT1 showed little impact on p27 and p21 (Supplementary Fig. 2d). Furthermore, silencing cyclin D1 abrogated GLUT1-mediated colony formation (Supplementary Fig. 2e).

### GLUT1 promotes iCCA cell migration and invasion in vitro

**Fig. 2:**
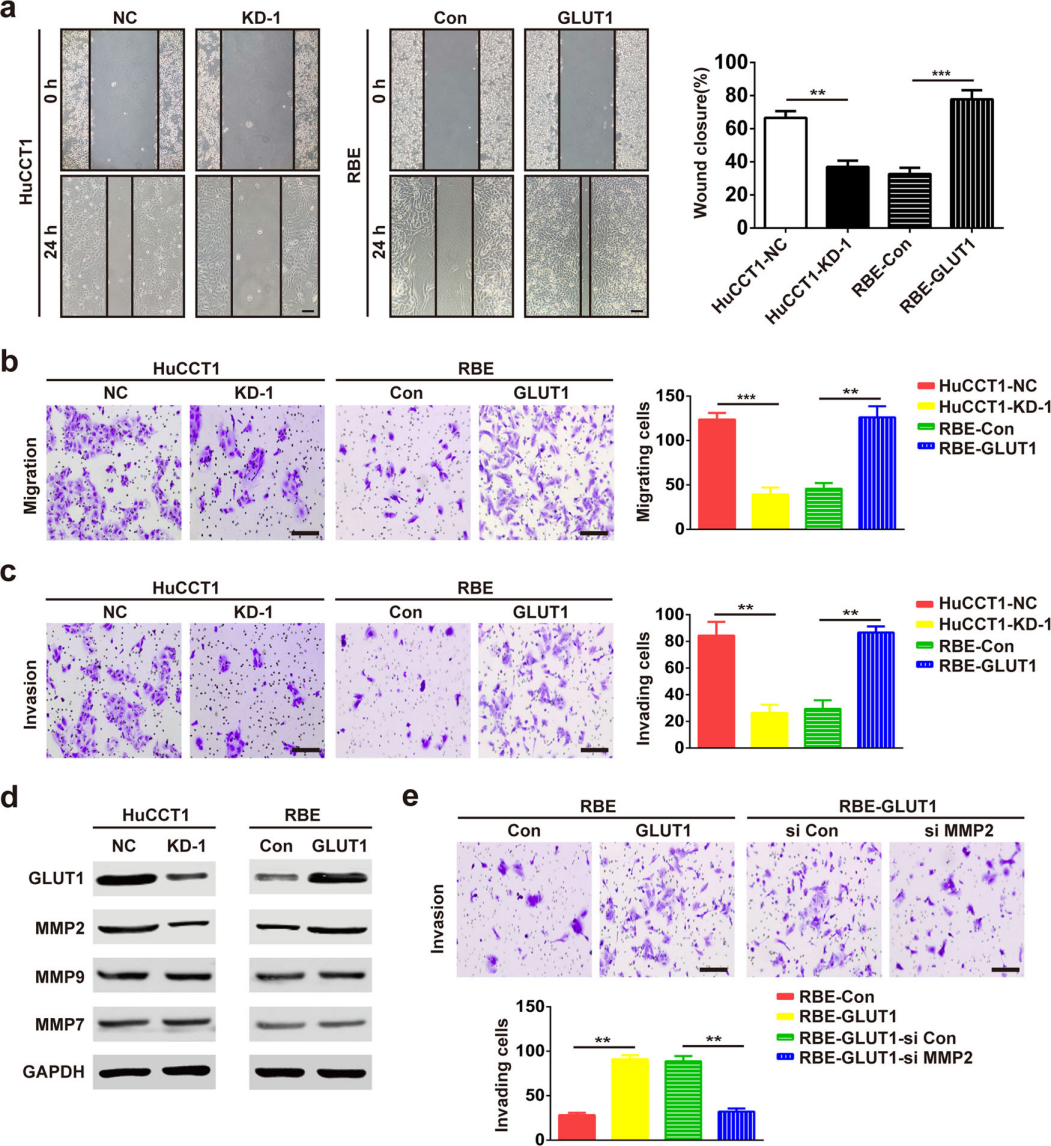
GLUT1 promotes iCCA cell migration and invasion in vitro. **a** The migration ability of iCCA cells was determined by wound-healing assays. The representative images were captured at 0 and 24 h. Bar = 300 μm. **b** and **c** Migration and invasion assays for the indicated cell lines after GLUT1 knockdown or overexpression. Bar = 100 μm. **d** Expression of MMP2, MMP7, and MMP9 was evaluated using western blot assays with the indicated processed cells and the corresponding control group. **e** MMP2 knockdown decreased the GLUT1-induced invasion of RBE cells. Bar = 100 μm. Data are means ± SD of three independent experiments. **P < 0.01; ***P < 0.001.

We investigated whether GLUT1 could affect iCCA cell motility. In the wound-healing assay, GLUT1 knockdown in HuCCT1 cells resulted in a slower closure of the scratched “wound” compared to that of the control. Conversely, GLUT1 overexpression enhanced the abilities of RBE cells to cover the scratched “wound” (Fig. 2a).

The transwell migration and invasion assays showed that the upregulation of GLUT1 significantly increased the migration and invasion abilities of RBE cells. In contrast, the inhibition of GLUT1 expression markedly reduced the migration and invasion abilities of HuCCT1 cells (Fig. 2b, c).

We then determined whether GLUT1 manipulates matrix metalloproteinases (MMPs), which are key metastasis-related proteins. GLUT1 inhibition significantly downregulated MMP2, whereas GLUT1 overexpression upregulated MMP2 (Fig. 2d). Nonetheless, GLUT1 had a minimal effect on MMP9 and MMP7 (Fig. 2d). Moreover, MMP2 knockdown abrogated GLUT1-mediated invasion (Fig. 2e).

### GLUT1 promotes growth and metastasis in vivo

**Fig. 3:**
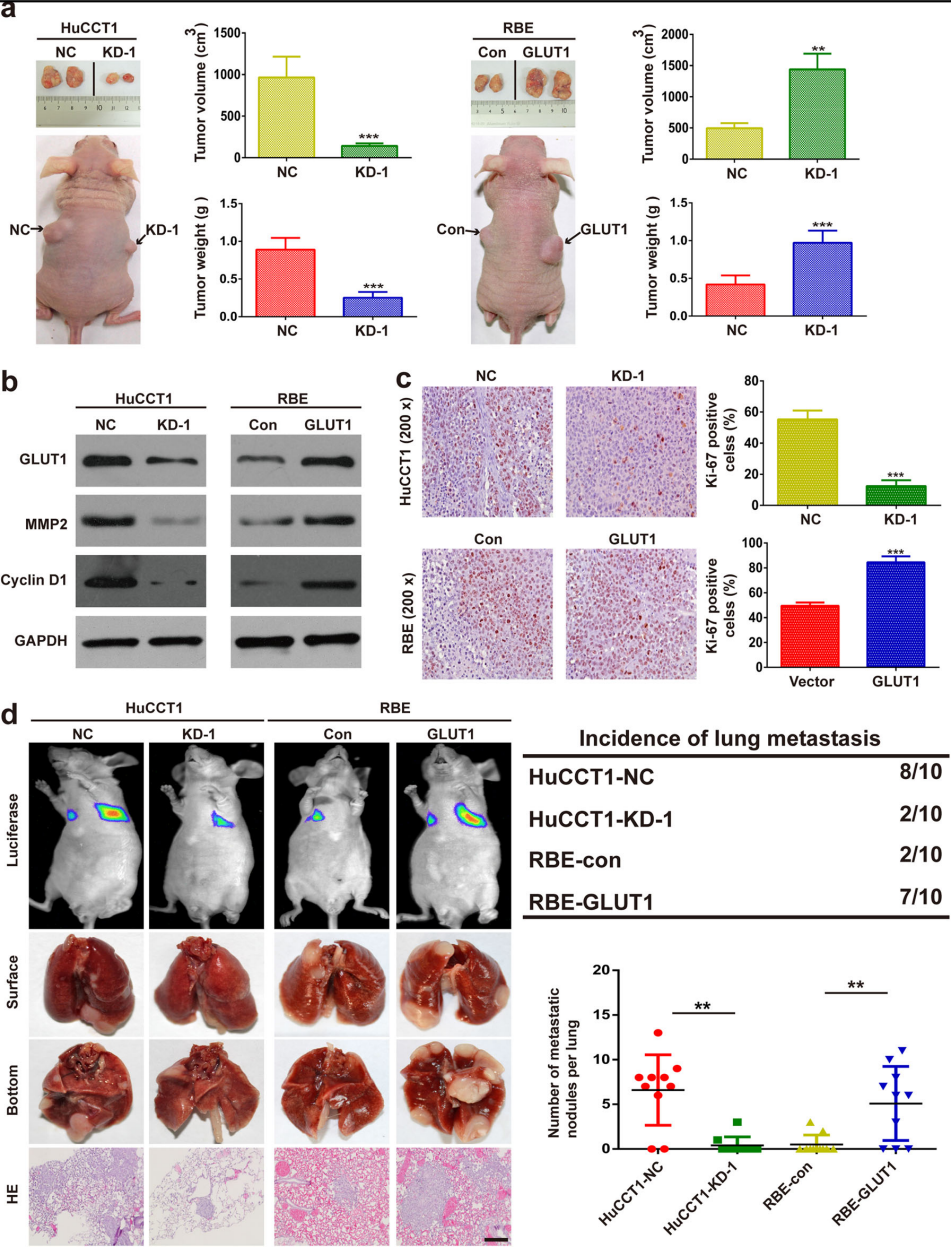
GLUT1 promotes growth and metastasis in vivo. **a** GLUT1 upregulation enhanced RBE cell xenograft tumor growth in nude mice, while GLUT1 knockdown reduced HuCCT1 growth. **b** The expression of MMP2 and cyclin D1 was evaluated using western blot assays with xenograft tissues. **c** IHC staining was used to detect Ki-67 expression in xenograft tissues. **d** Representative bioluminescence images, gross specimens, and hematoxylin and eosin (H&E) staining of the lung tumors are shown in the left panels. The number and incidence of nodules are displayed in the right panels. Bar = 100 μm. Data are means ± SD of three independent experiments. **P < 0.01; ***P < 0.001.

To determine whether GLUT1 could affect tumorigenesis in vivo, iCCA cell lines were injected subcutaneously into the flanks of nude mice. As shown in Fig. 3a, the inhibition of GLUT1 reduced the growth of xenograft tumors formed by the indicated iCCA cells, whereas the upregulation of GLUT1 promoted xenograft tumor growth. The expression of GLUT1, MMP2, and cyclin D1 in the tumor tissues was also evaluated with western blot assays, and the results obtained were similar to the results in vitro (Fig. 3b). The IHC results indicated that Ki-67 was significantly reduced in GLUT1-silenced xenograft tumors, while Ki-67 was significantly elevated in GLUT1-overexpressing tumors (Fig. 3c).

Furthermore, we evaluated the role of GLUT1 in tumor metastasis by injecting iCCA cells into nude mice via their tail veins and monitoring the presence of lung metastatic nodules. GLUT1 inhibition decreased the incidence of lung metastasis and the number of metastatic nodules, whereas GLUT1 overexpression increased the incidence and the number of metastatic nodules (Fig. 3d).

### GLUT1 inhibition sensitizes iCCA cells to gemcitabine in vitro and in vivo

**Fig. 4:**
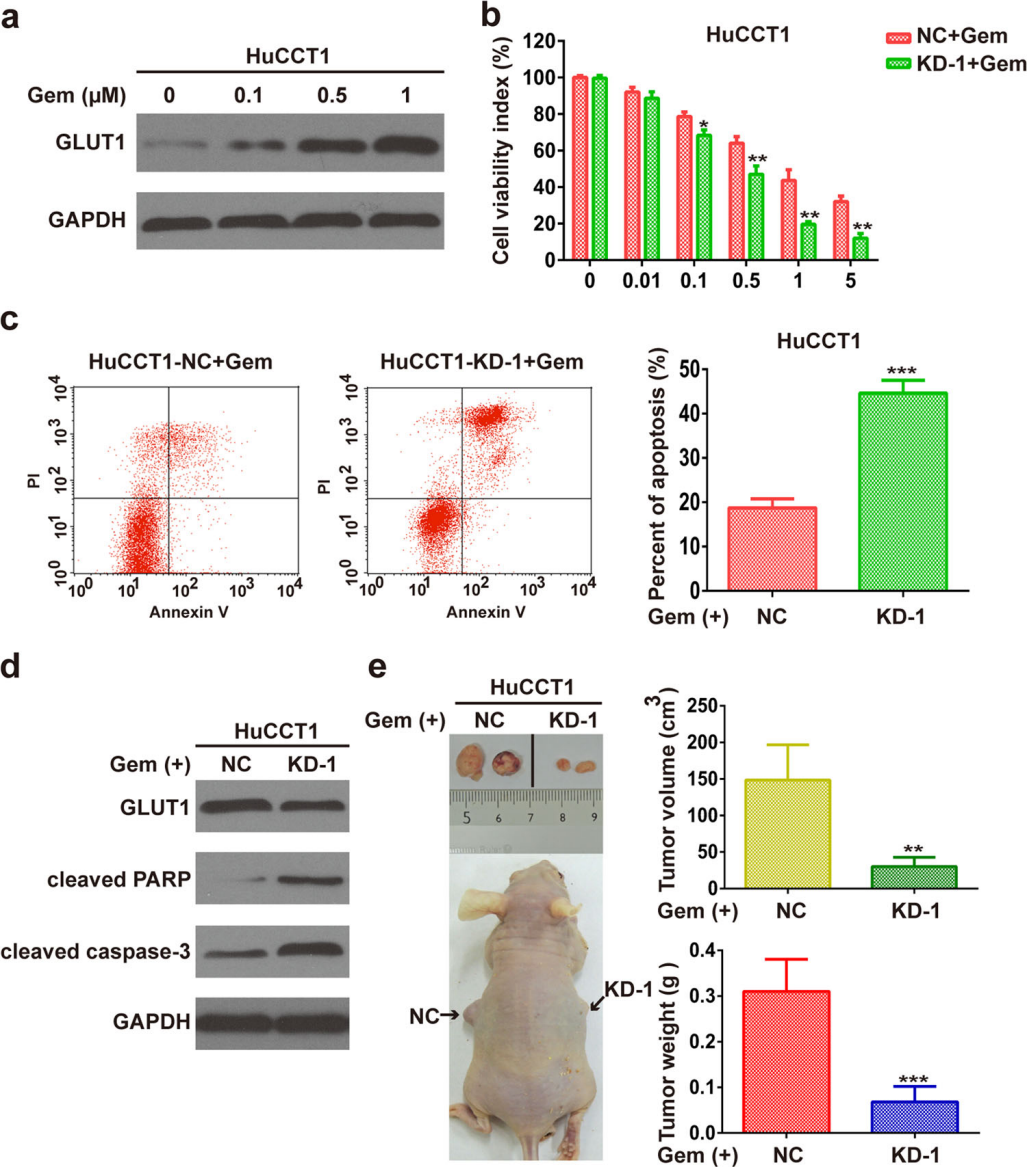
GLUT1 inhibition sensitizes iCCA cells to gemcitabine in vitro and in vivo. **a** Administration of gemcitabine induced the expression of GLUT1 in HuCCT1 cells in a dose-dependent manner. **b** CCK8 analyses show cell viability in gemcitabine-treated HuCCT1 cells with or without GLUT1 knockdown. **c** Flow cytometric analyses show cell apoptosis in gemcitabine-treated HuCCT1 cells with or without GLUT1 knockdown. **d** Western blot assays show the expression of cleaved caspase-3 and cleaved PARP in gemcitabine-treated HuCCT1 cells with or without GLUT1 knockdown. **e** GLUT1 knockdown increased the sensitivity of gemcitabine in vivo. Data are means ± SD of three independent experiments. **P < 0.01; ***P < 0.001.

We then investigated whether dysregulated GLUT1 had a potential effect on gemcitabine. The expression of GLUT1 protein increased with gemcitabine treatment in HuCCT1 cells (Fig. 4a). CCK8 assays revealed that silencing GLUT1 sensitizes HuCCT1 cells to gemcitabine (Fig. 4b). The half-maximal inhibitory concentration (IC50) was reduced in GLUT1-knockdown cells compared with that of the control cells (Supplementary Fig. 3). The knockdown of GLUT1 inhibited glycolysis, as indicated by decreased glucose consumption and lactate production (Supplementary Fig. 4). The inhibition of GLUT1 sensitizes HuCCT1 cells to gemcitabine-induced apoptosis as indicated by flow cytometric analysis (Fig. 4c). GLUT1 knockdown increased the gemcitabine-induced expression of cleaved caspase-3 and cleaved PARP (Fig. 4d). In vivo, GLUT1 knockdown increased the sensitivity of iCCA xenograft tumors to gemcitabine (Fig. 4e).

### GLUT1 is a direct target of miR-148a in iCCA

**Fig. 5:**
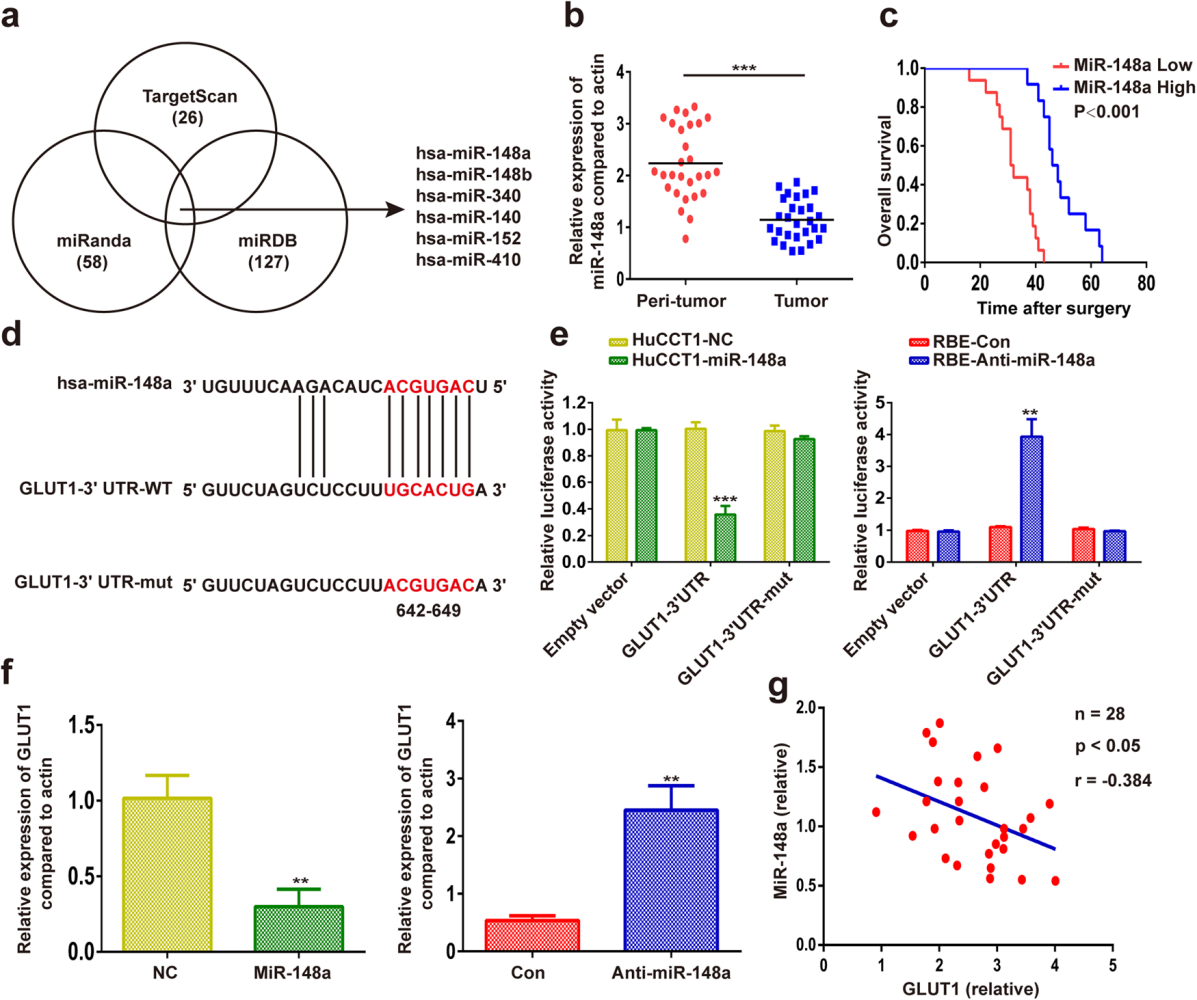
GLUT1 is a direct target of miR-148a in iCCA. **a** Analysis using TargetScan, miRanda, and miRDB revealed that several miRNAs might regulate GLUT1. **b** The miR-148a mRNA level was analyzed in 28 iCCA and paracancerous specimens using qRT-PCR. **c** Kaplan–Meier survival analyses were conducted to assess the influence of miR-148a on overall survival. **d** The binding site of miR-148a in the wild-type (WT) 3′-untranslated region (UTR) of GLUT1 and the corresponding mutant type were constructed. **e** A luciferase assay of GLUT1 3′-UTR and GLUT1 3′-UTR-mut reporters cotransfected with a miR-148a mimic or miR-148a inhibitor in the indicated cells. **f** MiR-148a overexpression or silencing decreased or increased GLUT1 mRNA in the indicated cells. **g** Correlation analysis indicated an inverse correlation between miR-148a and GLUT1 expression. Data are means ± SD of three independent experiments. *P < 0.05; **P < 0.01; ***P < 0.001.

To investigate the upstream regulatory mechanism of GLUT1 in iCCA, we assessed in silico the potential of miRNAs to bind to GLUT1 mRNA. By using TargetScan (http://www.targetscan.org/vert_72/), miRanda (http://www.microrna.org/microrna/home.do), and miRDB (http://www.mirdb.org/miRDB/) target prediction programs, six miRNAs were found to have a binding site in the 3ʹUTR region of the GLUT1 transcript (Fig. 5a). The expression of miR-148a, miR-340, and miR-152 was downregulated in iCCA samples among the six miRNAs, according to the results from the TCGA Pan-Cancer database (Supplementary Fig. 5). As the downregulation of miR-148a was more apparent than that of miR-340 and miR-152, we focused on miR-148a in subsequent experiments. Our results further confirmed that miR-148a expression was significantly lower in iCCA tissues than in matched adjacent non-tumor liver tissues (Fig. 5b). Patients with high miR-148a expression had longer overall survival than those with low miR-148a expression (Fig. 5c).

To confirm binding between miR-148a and GLUT1 3ʹ-UTR, we ran a luciferase reporter assay with either a wild-type or a mutated GLUT1 3ʹ-UTR-coupled luciferase reporter (Fig. 5d). Luciferase activity was suppressed by miR-148a in HuCCT1 cells transfected with wild-type GLUT1 3ʹ-UTR, whereas miR-148a inhibition enhanced luciferase reporter activity in RBE cells transfected with wild-type GLUT1 3′-UTR (Fig. 5e). Mutations of the 3ʹ-UTR sequence of GLUT1 reversed miR-148a suppression (Fig. 5e). The results from the RT-PCR revealed that GLUT1 expression was suppressed in miR-148a-expressing cells, whereas miR-148a inhibition upregulated the expression of GLUT1 (Fig. 5f). We then assessed whether the combination of GLUT1 and miR-148a could confer this benefit and found that GLUT1 and miR-148a expression levels were negatively correlated in iCCA samples (Fig. 5g).

### miR-148a inhibits iCCA growth and metastasis by targeting GLUT1

**Fig. 6:**
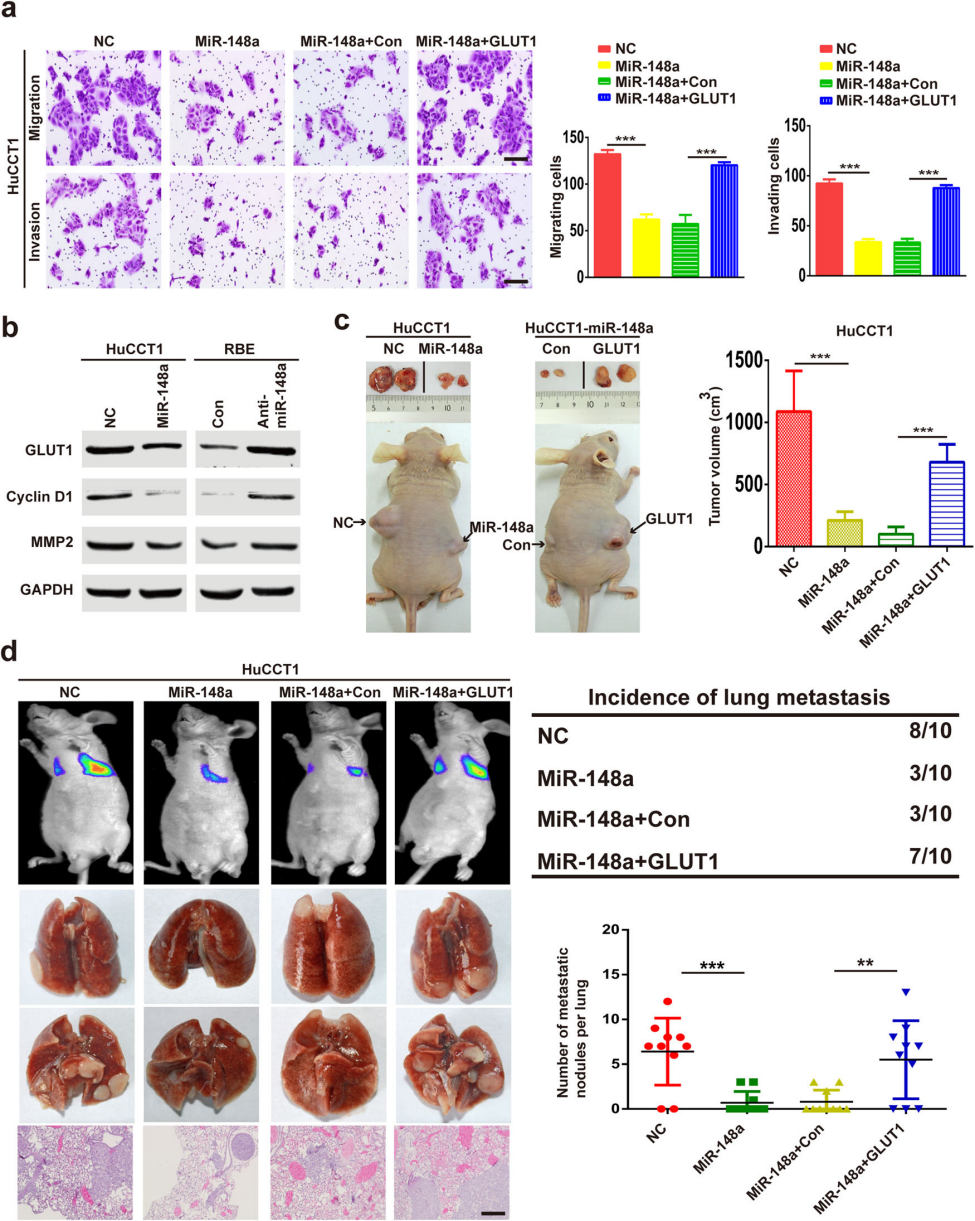
MiR-148a inhibits iCCA growth and metastasis by targeting GLUT1. **a** Migration and invasion assays were performed on HuCCT1 cells transfected with a negative control, miR-148a, miR-148a plus con, or miR-148a plus GLUT1. Bar = 100 μm. **b** MiR-148a overexpression or silencing decreased or increased GLUT1 protein levels in the indicated cells. **c** In vivo subcutaneous tumor growth assay of the indicated cells injected subcutaneously into the flanks of nude mice. Representative images and tumor volumes are shown. **d** In vivo metastasis assay of the indicated cells injected via the tail veins into nude mice. Data are means ± SD of three independent experiments. Bar = 100 μm. **P < 0.01; ***P < 0.001.

We investigated whether miR-148a affects iCCA growth and metastasis by regulating GLUT1 expression. Colony formation assays showed that miR-148a overexpression significantly reduced HuCCT1 cell growth, whereas GLUT1 upregulation enhanced HuCCT1 cell growth (Supplementary Fig. 6a). Conversely, miR-148a inhibition increased RBE cell growth, but GLUT1 repression restored RBE cell growth (Supplementary Fig. 6a). The transwell assay showed that miR-148a overexpression significantly reduced HuCCT1 cell migration and invasion, whereas GLUT1 upregulation enhanced HuCCT1 cell migration and invasion (Fig. 6a). Conversely, miR-148a inhibition increased RBE cell migration and invasion, but GLUT1 repression restored RBE cell migration and invasion (Supplementary Fig. 6b). The results of the western blot assays revealed that miR-148a overexpression inhibited the expression of GLUT1, cyclin D1, and MMP2 in HuCCT1 cells. On the other hand, miR-148a inhibition upregulated GLUT1, cyclin D1, and MMP2 in RBE cells (Fig. 6b). To determine whether these results were reproducible in vivo, iCCA cells were subcutaneously injected into the flanks or injected into the tail veins of nude mice. Tumor growth and lung tumor metastasis were significantly decreased by miR-148a overexpression, whereas GLUT1 upregulation increased tumor growth and lung tumor metastasis (Fig. 6c, d, and Supplementary Fig. 7).

### Targeting GLUT1 is effective in suppressing tumor growth in an iCCA patient-derived xenograft (PDX)

An iCCA PDX model was established to investigate the treatment efficacy of WZB117, a GLUT1 inhibitor. The expression of GLUT1 in the tumors of iCCA patients was first detected (Supplementary Fig. 8a). We then performed experiments in two selected PDX models with low (PDX#1) and high (PDX#4) GLUT1 levels. The results indicated that WZB117 inhibited the tumor growth of the PDX#4 models but did not inhibit tumor growth in the PDX#1 group (Supplementary Figs. 8b and 9). Accordingly, the results of western blot assays showed that the treatment of tumors with WZB117 resulted in a significant decrease in GLUT1, cyclin D1, and MMP2 in PDX#4 models (Supplementary Fig. 8c). No marked changes were observed in either the body, liver, or spleen weights of the mice in our experiments (Supplementary Fig. 8d–f).

## Discussion

In the present work, we found that GLUT1 was commonly overexpressed in human iCCA, which may be partially due to the downregulation of miR-148a. The upregulated expression of GLUT1 and the downregulated expression of miR-148a were related to shorter overall and disease-free survival. In addition, GLUT1 promoted iCCA growth, metastasis, and chemoresistance to gemcitabine. Importantly, we found that targeting GLUT1 with WZB117 effectively inhibited tumor growth in an iCCA PDX model. To our knowledge, this is the first study to intensively evaluate the biological relevance of the miR-148a–GLUT1 axis in the progression and drug resistance of iCCA.

The aberrant expression of GLUT1 has been previously reported in iCCA tissues^14^. The data from our RT-PCR, western blot, and IHC assays confirmed the significant upregulation of GLUT1 expression in iCCA tissues. The incidence of lymphatic metastasis was higher in the GLUT1 high-expression group than in the GLUT1 low-expression group. As metastasis is an important factor leading to the death of iCCA patients, we further explored whether the expression of GLUT1 is involved in patient survival. Results showed that high GLUT1 expression was associated with shorter overall survival, as compared to that with low GLUT1 expression. Furthermore, our gain-of-function and loss-of-function experiments clearly suggested the growth-promoting and metastasis-promoting role of GLUT1 in iCCA. Although the involvement of GLUT1 in cancer growth and metastasis has been documented in several human malignancies^22–25^, the underlying mechanisms remain unclear.

MMPs destroy the local tissue architecture and basement membranes thus allowing tumor invasion and metastasis^26^. We found that GLUT1 positively regulated the expression of MMP2 but had a minimal effect on the expression of MMP9 and MMP7. Furthermore, the inhibition of GLUT1 arrested the iCCA cells at the G1 phase by downregulating the expression of D1, which plays a crucial role in the regulation of the cell cycle during the G1/S phase transition^27^. Therefore, GLUT1 is a candidate oncogene for iCCA risk prognostication and therapy.

In an attempt to explore the molecular basis for GLUT1 overexpression in iCCA, we evaluated an in silico database and found that miR-148a may be an upstream regulator of GLUT1. As expected, miR-148a is downregulated in iCCA tissues compared to adjacent non-tumor liver tissues. MiR-148a inhibits GLUT1 endogenous expression and directly targets GLUT1 by complementarily binding to its 3′-UTR. Rescue experiments indicated that GLUT1 knockdown partly mimicked the inhibitory function of miR-148a, while GLUT1 overexpression attenuated the effects of miR-148a overexpression. Although several mechanisms for the upregulation of GLUT1 have been revealed^28^, the results of the present study cannot rule out the possible involvement of those mechanisms that contribute to GLUT1 overexpression in iCCA. Nevertheless, we deduced that the downregulation of miR-148a partially accounts for the upregulation of GLUT1 in iCCA.

Gemcitabine is a pyrimidine analog that has been widely used as a first-line chemotherapeutic drug in the treatment of iCCA^29^. Unfortunately, patients with iCCA are prone to be resistant to gemcitabine, leading to a recurrence of iCCA. This acquired drug resistance remains the most common and primary clinical cause of chemotherapy failure^30^. Recently, several studies have revealed that the Warburg effect is involved in gemcitabine resistance and that gemcitabine resistance was overcome by effectively targeting the Warburg effect^31^. In the present study, we found that the inhibition of GLUT1 sensitizes iCCA cells to gemcitabine both in vitro and in vivo. These results showed that GLUT1 participates in gemcitabine resistance.

We also provided a pre-clinical evaluation of WZB117 in iCCA and built a case for the development of a clinical trial for WZB117 as a first-line or second-line therapy for iCCA. We showed that the administration of WZB117 suppressed tumor growth in an iCCA PDX model. Thus, targeting GLUT1 in combination with gemcitabine seems to be a promising novel therapeutic strategy to improve the treatment efficacy of iCCA patients.

The present study revealed that the abnormal expression of the miR-148a–GLUT1 axis plays an indispensable role in the growth, metastasis, and chemoresistance of iCCA. These findings suggest that the miR-148a–GLUT1 axis has clinical value for the development of novel therapeutic strategies for iCCA patients and warrants continued investigation in this regard.

## Materials and methods

### Patients and tissue samples

A retrospective cohort of 60 iCCA patients was included in the present study. All patients had undergone routine surgical procedures between 2004 and 2013 at the First Affiliated Hospital of Harbin Medical University, Harbin, China. The inclusion criteria were as follows: primary diagnosis of iCCA between 2004 and 2013 (at least 5 years of potential follow-up); no previous diagnosis of carcinoma; no evidence of disease within 1 month of primary surgery. Patients who received neoadjuvant treatment before primary surgery were excluded. RNA or protein was extracted from their resected tissues and analyzed by RT-PCR or western blot assays, respectively. The pathological diagnosis of iCCA was performed according to World Health Organization (WHO) criteria. Grades of differentiation were evaluated using the classification proposed by Edmondson and Steiner. All samples used in this study were approved by the Committees for the Ethical Review of Research at the First Affiliated Hospital of Harbin Medical University, Harbin, China. Informed consent was received from each participant before surgery in this study.

### Cell culture, reagents, and lentivirus

The human iCCA cell line RBE was obtained from Shanghai Bioleaf Biotech Co. Ltd. (Shanghai, China). The human iCCA cell line HuCCT1 was kindly provided by the Cancer Cell Repository of Tohoku University in Sendai, Japan. The two iCCA cell lines were cultured in RPMI 1640 medium (Gibco, Invitrogen Company, Grand Island, NY) supplemented with 10% fetal bovine serum (FBS; Gibco) and 100 U/mL penicillin (Gibco) at 37 °C under a 5% CO_2_ atmosphere. All cell lines were identified by short tandem repeat typing. The primary antibodies against MMP2 (#40994), MMP7 (#3801), MMP9 (#13667), cyclin D1 (#55506), p27 (#3686), p21 (#2947), and Ki-67 (#9449) were purchased from Cell Signaling Technology (Danvers, MA). The primary antibodies against GLUT1 were obtained from Abcam (Cambridge, UK, ab128033) and Cell Signaling Technology (Danvers, MA, #12939). WZB117 was purchased from Sigma-Aldrich Corp. (St. Louis, MO). The lentiviral vectors overexpressing human GLUT1 and miR-148a (LV-GLUT1 and miR-148a) and their corresponding knockdown forms (LV-sh GLUT1 and LV-anti-miR-148a) were constructed and synthesized by Shanghai GeneChem Corporation (Shanghai, China). Empty vectors were used as the corresponding controls.

### CCK8 assay

Cell proliferation was assessed using the CCK8 Kit (Dojindo, Kumamoto, Japan). The cells were seeded in a 96-well plate at a density of 3000 per well, and their viability was determined 48 h later. The cell medium was replaced with 100 μL of complete medium supplemented with 10 μL CCK8, and the cells were incubated at 37 °C with 5% CO_2_ for 2 h. Absorbance values (OD) were measured at 450 nm in a microplate reader (SpectraMax M2, Molecular Devices, Sunnyvale, CA).

### Growth curve and colony formation assays

To plot the growth curve, the iCCA cells were seeded in six-well plates at a density of 1 × 10^4^/well. The viable cells were counted daily for 6 days. For the colony formation assay, 1000 iCCA cells were cultured in six-well plates for 2–3 weeks. The colonies were then stained with 0.05% crystal violet for 30 min and counted.

### Cell cycle analysis

Cells (4 × 10^5^) were fixed in 70% ethanol for 1 h at 4 °C. Thereafter, the cells were washed twice with phosphate-buffered saline (PBS), and 10 mg/mL RNase A was added. Propidium iodide was then added to the tubes to obtain a final concentration of 0.05 mg/mL, and the samples were incubated at 4 °C for 30 min in a dark environment. The result was analyzed by flow cytometry.

### Apoptosis assay

The iCCA cells (3 × 10^5^/well) were cultured in six-well plates and were collected and washed twice with ice-cold PBS. Apoptosis was investigated by flow cytometry using the Annexin V-PE Apoptosis Kit (Becton Dickinson, San Diego, CA) following the protocol of the manufacturer.

### Wound healing

The cells were cultured in a six-well plate and allowed to grow to confluence. They were washed 3× in medium, scratched with a 200-μL pipette tip, and microphotographed at 0 and 24 h under a Nikon Eclipse TS100 microscope (Nikon, Tokyo, Japan).

### Cell migration and invasion assays

For the transwell migration assay, 2.5 × 10^4^ cells were seeded into the upper chamber in serum-free media and incubated at 37 °C for 24 h. Thereafter, migration toward the normal media was determined. Cell invasion assays were performed in 24-well dishes (Nuclepore^TM^; GE Healthcare, Chicago, IL) whose chambers were lined with membranes that were precoated with Matrigel (BD Biosciences, San Jose, CA). The assays were run at 37 °C for 36 h. Cells migrating through the membrane were fixed in methanol, stained with 0.5% crystal violet in methanol, and counted under a light microscope.

### Dual-luciferase assay

The indicated cells were plated and cultured in triplicate for 24 h and co-transfected with either GLUT1 3′-UTR clones or mutant clones with the pRL-TK Renilla plasmid. After 48 h, luciferase activity in the transfected cells was measured with the Dual-Luciferase Reporter Assay Kit (Promega, Madison, WI) according to the protocol of the manufacturer.

### Western blot

A western blot assay was performed as has been previously described^32^. In brief, whole-cell extracts were sonicated in lysis buffer and homogenized. Samples containing 30–50 µg total protein were resolved on 8–12% polyacrylamide–SDS gels and electrophoretically transferred to polyvinylidene difluoride (PVDF) membranes. The membranes were blocked with 5% skim milk, incubated with primary antibody, then incubated with an alkaline phosphatase-conjugated secondary antibody. Protein bands were detected with a chemiluminescence kit (Roche Diagnostics Corp., Indianapolis, IN, USA).

### RT-PCR analysis

Total RNA from cells or human tissues was prepared with the RNeasy Kit (Qiagen, Valencia, CA). The cDNA was prepared with TaqMan® Reverse Transcription Reagents (Applied Biosystems, Grand Island, NY). Human GLUT1 and GAPDH probe were purchased from Applied Biosystems. A TaqMan real-time PCR was run with a TaqMan PCR mixture (Applied Biosystems).

MicroRNAs were prepared with an ultrapure miRNA isolation kit (Roche Diagnostics Corp., Indianapolis, IN). Real-time PCR was performed with a TaqMan® MicroRNA Reverse Transcription Kit (Applied Biosystems). All human miRNA probes were purchased from Applied Biosystems. MicroRNA real-time PCR was run with a TaqMan® Universal Master Mix II, No UNG (Applied Biosystems).

### Animal experiments

All mice were obtained from the Animal Center Laboratory of the Chinese Academy of Sciences, Shanghai, China. The experimental protocol was reviewed and approved by the Committee on the Use of Live Animals in Teaching and Research of the Harbin Medical University, Harbin, China.

For subcutaneous tumor models, 3 × 10^6^ iCCA cells were resuspended in 200 µL PBS and injected subcutaneously into the flanks of 5-week-old male BALB/c nude mice. Tumor volumes were measured using an electronic caliper and calculated using the following formula: ${\mathrm {Volume}}\left( {\mathrm {cm}^{3}} \right) = {\it{L \times W}}^2 \times \frac{\pi }{6}$^,^ where *L* and *W* represent the largest and smallest diameters, respectively. Tumor formation was observed in the mice for 5 or 6 weeks. Then, the mice were sacrificed, and their tumors were excised for use in subsequent experiments.

For the chemosensitivity assay, 2 × 10^6^ iCCA cells were resuspended in 200 µL PBS and injected subcutaneously into the flanks of 5-week-old male BALB/c nude mice. Gemcitabine administration began 10 days after inoculation (25 mg/kg, twice weekly). The mice were observed over 5 weeks for tumor formation. The mice were then sacrificed, and their tumors were excised for use in subsequent experiments.

For the PDX model, PDXs were implanted in 5-week-old male BALB/c nude mice as previously described^33,34^. Briefly, after mice were anesthetized with 1.5–3% isoflurane, an incision was made in the flank area in the middle of the thigh line and enlarged to 0.5 cm. Following the implantation of a tissue fragment (1 mm × 1 mm × 1 mm) into a pocket created under the flank fat pad, the incision was sealed with Vetbond™ (3 M). Drug administration began when the tumors reached 5 mm × 5 mm (*L* × *W*) in size. The mice were randomized for treatment with either PBS/DMSO (1:1, v/v) or WZB117 (10 mg/kg, once daily) dissolved in PBS/DMSO solution (1:1, v/v). Tumor formation was observed in the mice for 6 weeks. At the end of the treatment period, the mice were sacrificed and their tumors were excised for use in subsequent experiments.

### IHC

IHC staining and scoring were performed as has been previously described^35^. GLUT1 was incubated with the specimens and biotinylated secondary antibody was applied at a dilution of 1:200 before HRP application (Vectastain ABComplex; Vector Laboratories, Inc., Burlingame, CA) and produced brown positive staining. Briefly, the IHC reaction was scored by multiplying the proportion of positive tumor cells (PP: 0 = no positive tumor cells; 1 = <10%; 2 = 10–50%; 3 = 51–80%; 4 = >80% positive tumor cells) by their predominant degree of staining (SI: 0 = negative; 1 = weak; 2 = moderate; 3 = strong staining). IHC staining intensity in the tumor cells was scored independently by two pathologists, and the average was taken as the final score. The median score of GLUT1 was used as the cutoff to segregate the patients into either the high (≥median) or low (<median) group.

### Lactate assay

For this, 4 × 10^5^ cells/well were seeded in six-well plates. Cells were homogenized with lactate assay buffer 48 h after seeding. Lactate quantification was performed using a commercially available lactate assay kit (Sigma, Saint Louis, USA) in a 96-well plate as per the manufacturer’s instruction. Lactate production was measured with a plate reader (BioTek Eon) at an optical density of 570 nm and lactate was quantified in accordance with the manufacturer’s protocol.

### Glucose uptake assay

First, 4 × 10^5^ cells/well were seeded in six-well plates. Cells were homogenized with glucose assay buffer 48 h after seeding. The quantification of glucose levels was performed using a commercially available glucose assay kit (Abcam, Melbourne, Australia) in a 96-well plate, as per the manufacturer’s protocol. Glucose uptake was measured with a plate reader (BioTek Eon) at an optical density of 570 nm.

### Statistical methods

Statistical analyses were performed with GraphPad Prism v. 4.02 (San Diego, CA) or with SPSS v. 16.0 (IBM Corp., Armonk, NY). Data are expressed as means ± standard deviation (SD). Analysis of variance (ANOVA), a Student’s *t*-test, and a Wilcoxon test were used to evaluate statistical significance. Overall survival and disease-free survival were compared by the Kaplan–Meier method, and significance was determined by a log-rank test. Correlations were calculated by a Pearson correlation analysis. A *P*-value < 0.05 indicated statistical significance.

## Supplementary information

Supplementary information

Figure S1

Figure S2

Figure S3

Figure S4

Figure S5

Figure S6

Figure S7

Figure S8

Figure S9
