# Supplementary material for: Dysregulation of the miR-148a–GLUT1 axis promotes the progression and chemoresistance of human intrahepatic cholangiocarcinoma
Source: Oncogenesis. 2020 Feb 13;9(2):19. doi: 10.1038/s41389-020-0207-2 (PMC7018977; doi:10.1038/s41389-020-0207-2)
Supplement: Supplementary file 1 — Supplementary information [file 41389_2020_207_MOESM1_ESM.docx]

**Figure S1.** The expression of GLUT1 was either upregulated or downregulated. After lentivirus transfection, GLUT1 was overexpressed or knocked down in iCCA cells. Data are means ± SD of three independent experiments. * * *P* < 0.01. * * * *P* < 0.001.

**Figure S2.** Inhibition of GLUT1 suppresses iCCA proliferation and cell cycle progression *in vitro*. (a) Proliferation was analyzed by growth curve assays of the indicated iCCA cells. (b) The cell cycle distribution after GLUT1 inhibition or overexpression in the indicated iCCA cells. (c) The representative images of the colony formation assays are shown in the left panels; the number of foci was counted as shown in the right panels. (d) Expression of cyclin D1, p27, and p21 was evaluated using western blot assays with the indicated processed cells and the corresponding control group. (e) Cyclin D1 knockdown decreased the GLUT1-induced colony formation of RBE cells. Data are means ± SD of three independent experiments. * *P* < 0.05. * * *P* < 0.01. * * * *P* < 0.001.

**Figure S3.** The IC50 of gemcitabine-treated HuCCT1 cells with or without GLUT1 knockdown.

**Figure S4.** Glucose consumption and lactate production in gemcitabine-treated HuCCT1 cells with or without GLUT1 knockdown.

**Figure S5.** The expression of the six indicated miRNAs in eight adjacent liver tissues and 33 iCCA tissues, according to the results from the TCGA Pan-Cancer database. Data are means ± SD of three independent experiments. * *P* < 0.05. * * * *P* < 0.001.

**Figure S6.** MiR-148a inhibits iCCA growth and metastasis by targeting GLUT1 (a) MiR-148a overexpression repressed HuCCT1 cell growth, whereas GLUT1 stimulated cell proliferation, as determined by the colony formation assay. MiR-148a inhibition promoted RBE cell growth, whereas GLUT1 knockdown inhibited cell proliferation as determined by the colony formation assay. (b) Migration and invasion assays performed on RBE cells transfected with control, anti-miR-148a, anti-miR-148a plus negative control, or anti-miR-148a plus shRNA1. Bar = 100 μm. Data are means ± SD of three independent experiments. * * *P* < 0.01. * * * *P* < 0.001.

**Figure S7.** *In vivo* subcutaneous tumor growth assay of the indicated cells injected subcutaneously into the flanks of nude mice. Tumor weights are shown. Data are means ± SD of three independent experiments. * * * *P* < 0.001.

**Figure S8.** Targeting GLUT1 is effective in suppressing tumor growth in an iCCA patient-derived xenograft (PDX) model. (a) Expression of the GLUT1 protein in iCCA patients. (b) WZB117 inhibited xenograft tumor growth in PDX#4 models. (c) The expression of MMP2 and cyclin D1 was evaluated using western blot assays with xenograft tissues. (d-f) There was no apparent change in either the body, liver, or spleen weights of the nude mice in either the WZB117-treatment or control group. Data are means ± SD of three independent experiments. * * *P* < 0.01. * * * *P* < 0.001.

**Figure S9.** WZB117 showed no effect on the PDX#1 models.
