# Supplementary figures and images for: Dysregulation of the miR-148a–GLUT1 axis promotes the progression and chemoresistance of human intrahepatic cholangiocarcinoma
Source: Oncogenesis. 2020 Feb 13;9(2):19. doi: 10.1038/s41389-020-0207-2 (PMC7018977; doi:10.1038/s41389-020-0207-2)

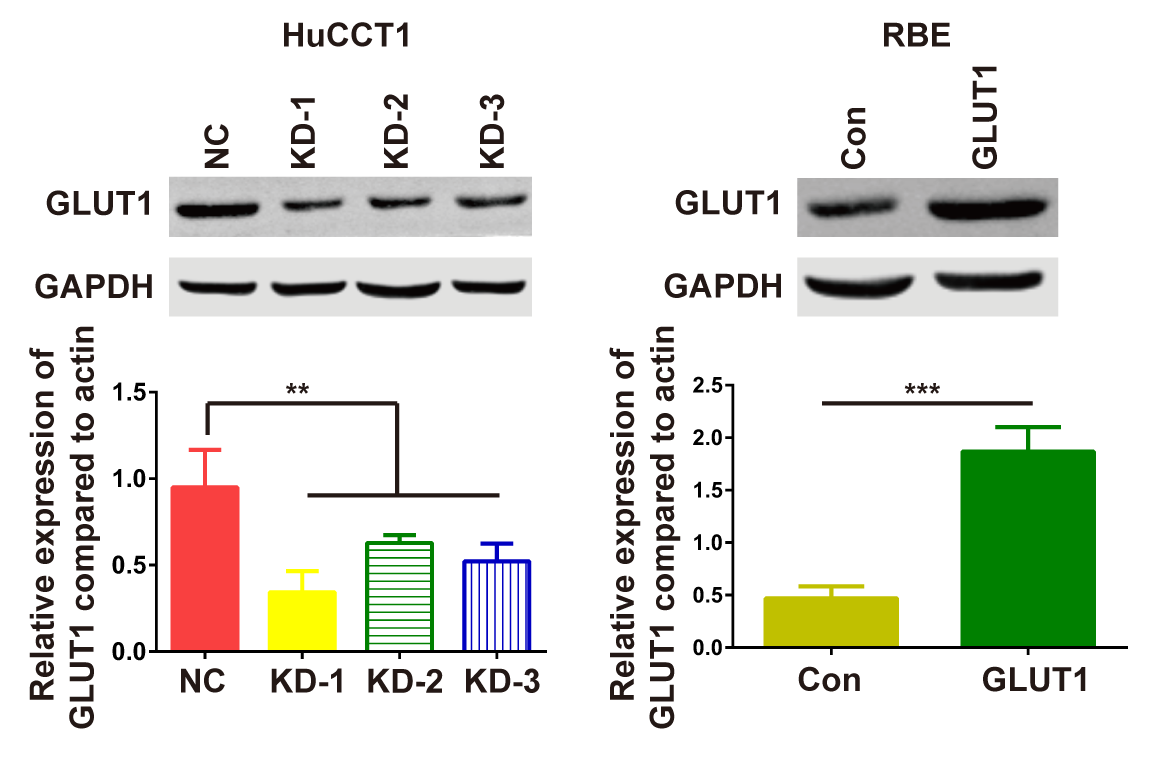

Supplement: Supplementary file 2 — Figure S1 [file 41389_2020_207_MOESM2_ESM.tif]

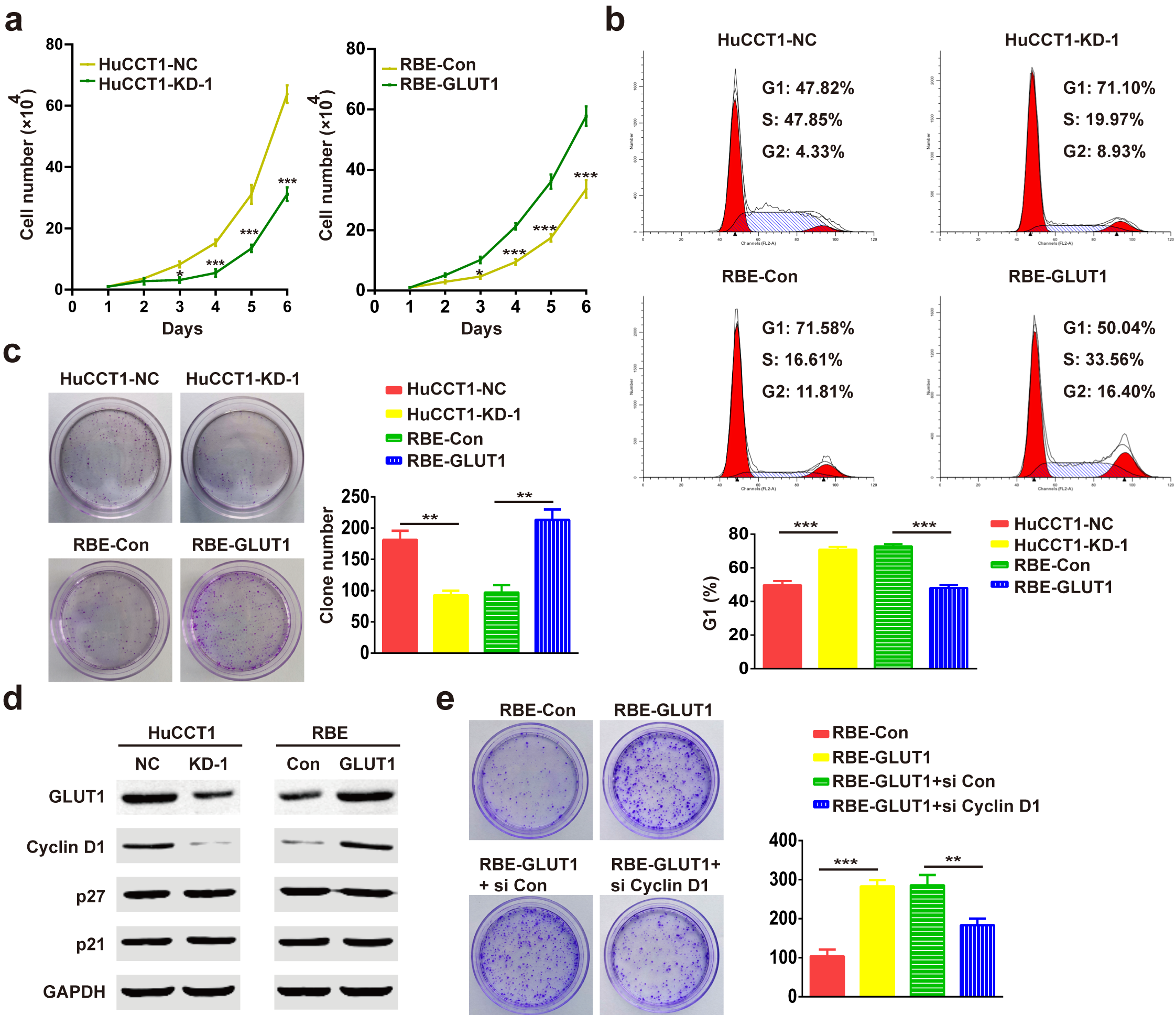

Supplement: Supplementary file 3 — Figure S2 [file 41389_2020_207_MOESM3_ESM.tif]

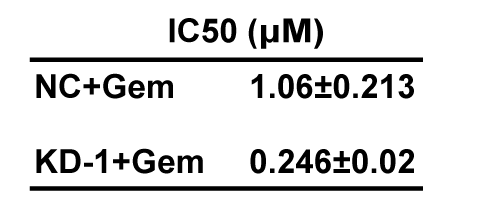

Supplement: Supplementary file 4 — Figure S3 [file 41389_2020_207_MOESM4_ESM.tif]

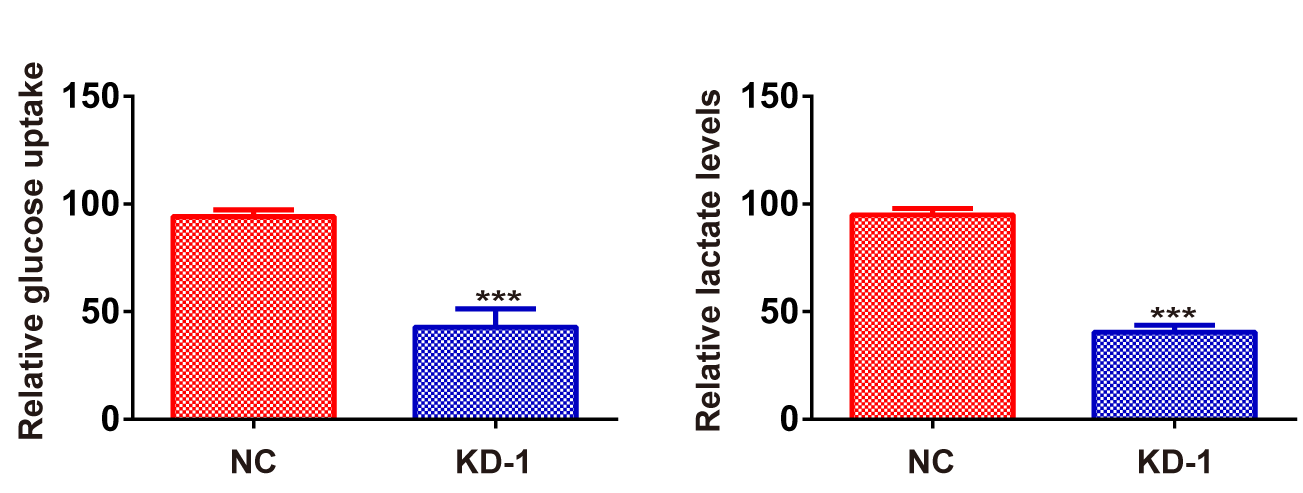

Supplement: Supplementary file 5 — Figure S4 [file 41389_2020_207_MOESM5_ESM.tif]

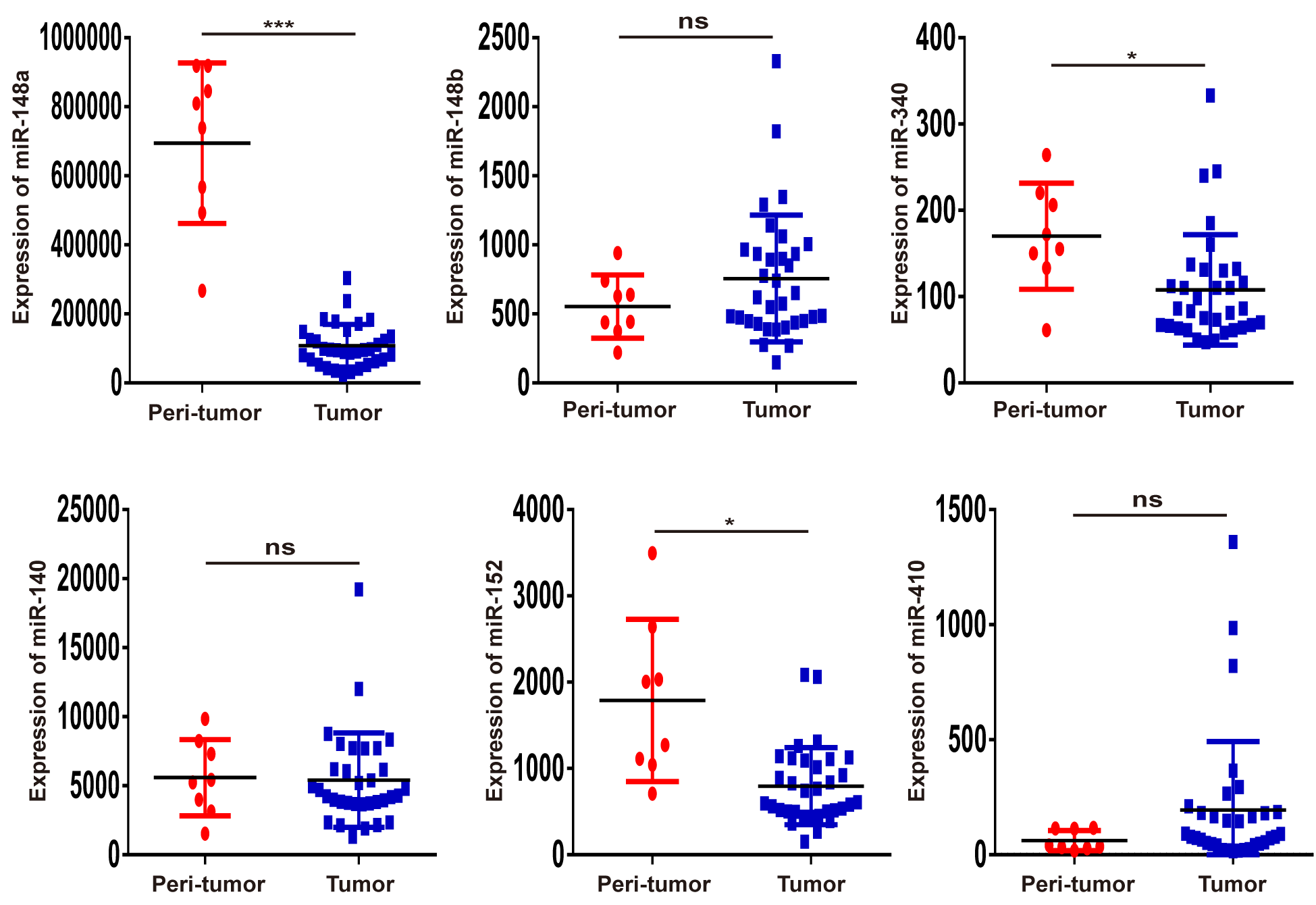

Supplement: Supplementary file 6 — Figure S5 [file 41389_2020_207_MOESM6_ESM.tif]

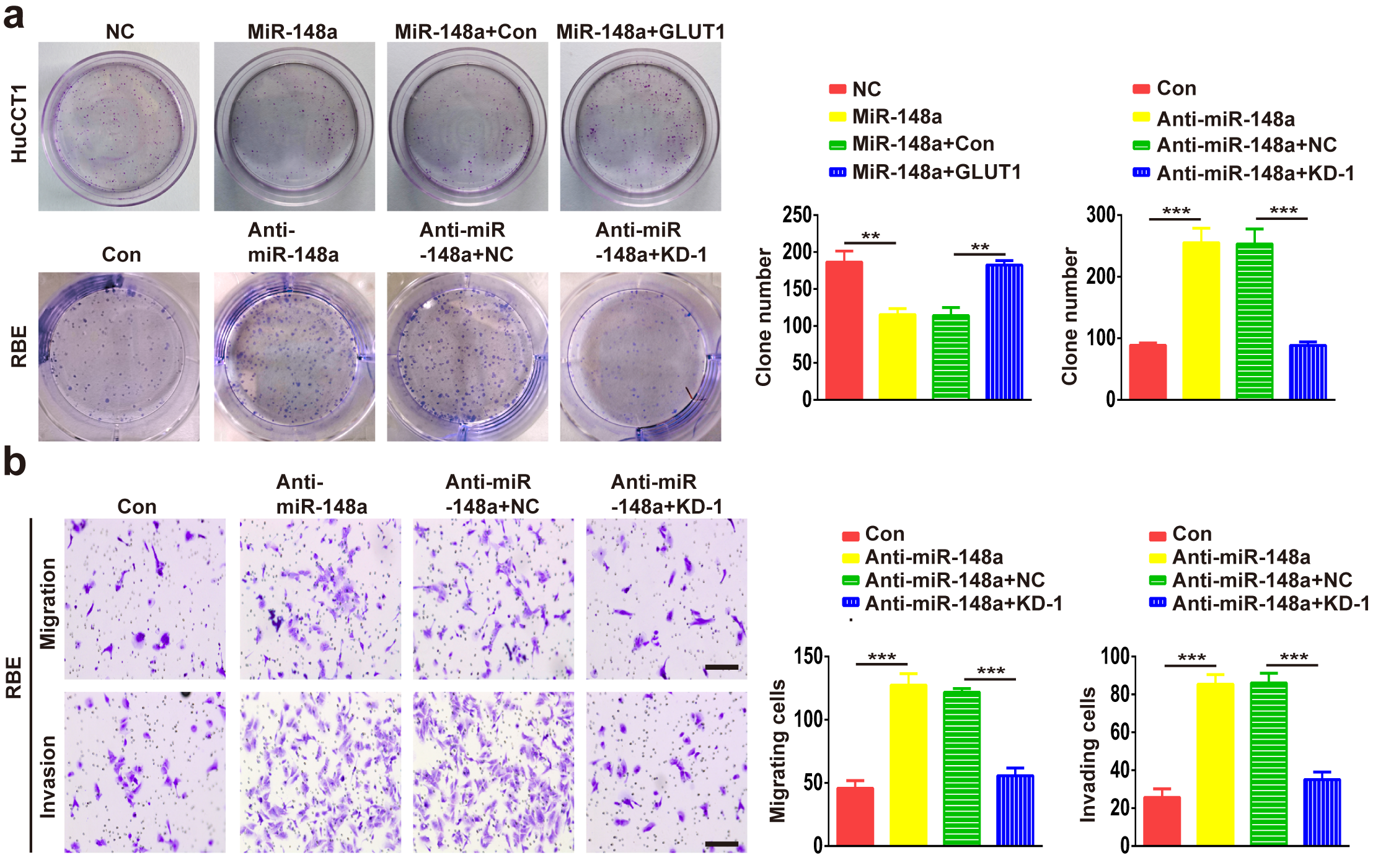

Supplement: Supplementary file 7 — Figure S6 [file 41389_2020_207_MOESM7_ESM.tif]

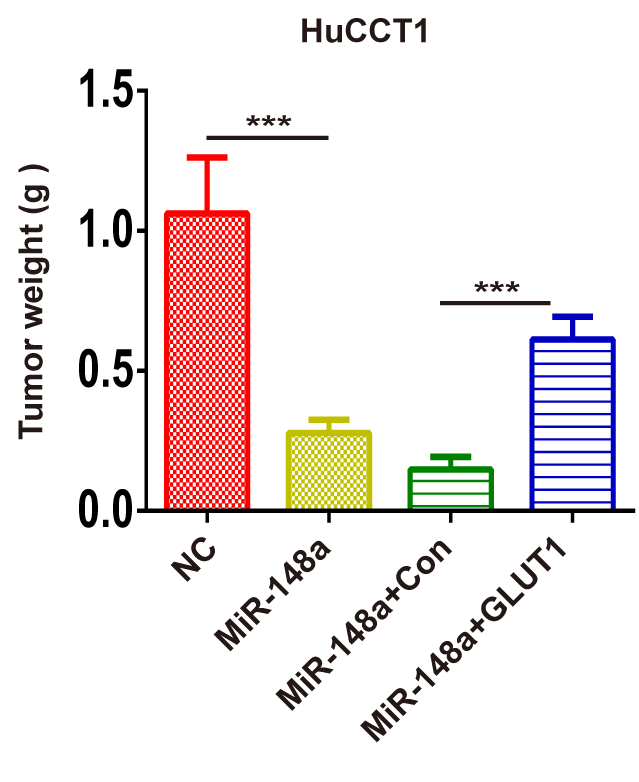

Supplement: Supplementary file 8 — Figure S7 [file 41389_2020_207_MOESM8_ESM.tif]

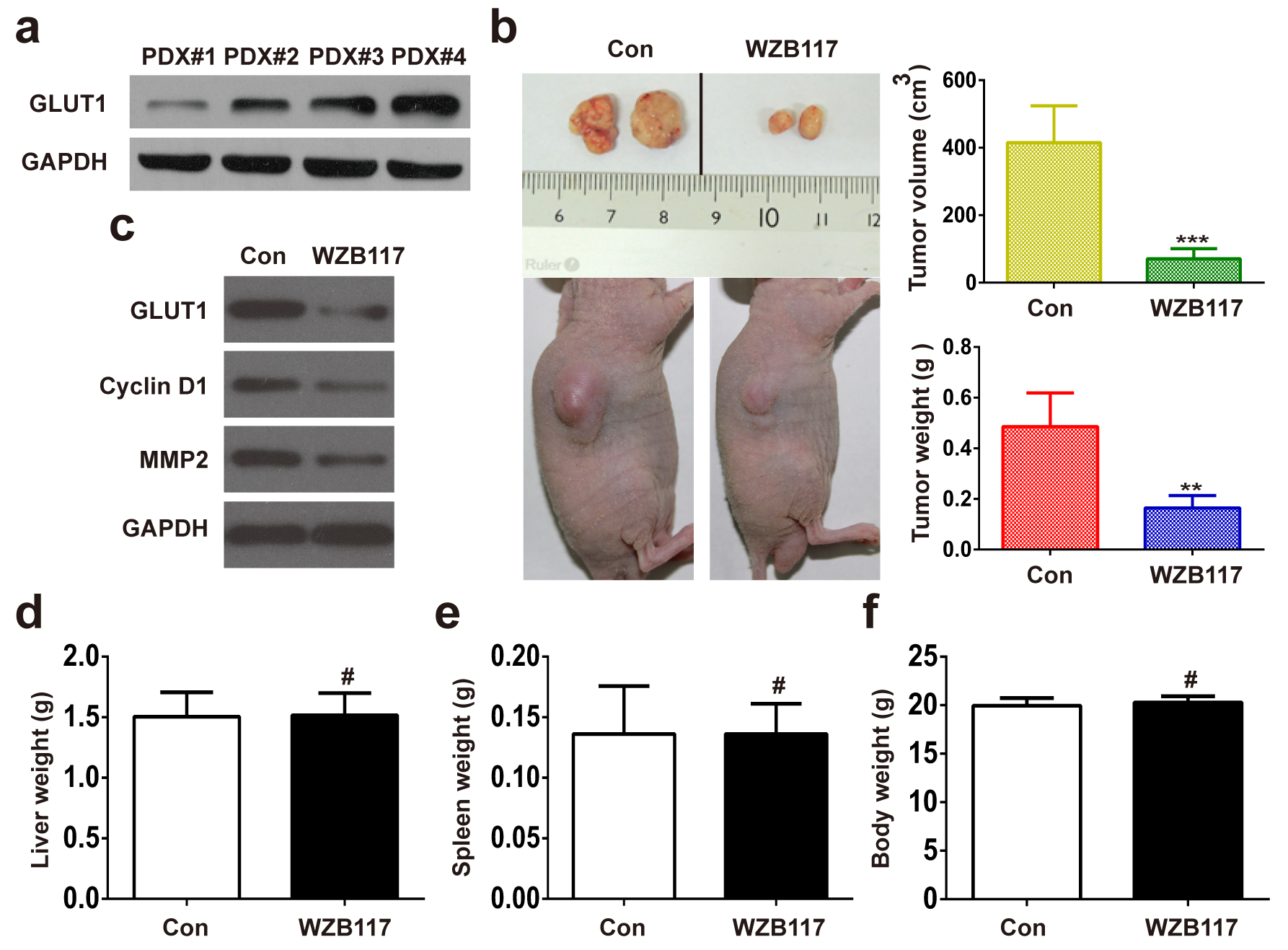

Supplement: Supplementary file 9 — Figure S8 [file 41389_2020_207_MOESM9_ESM.tif]

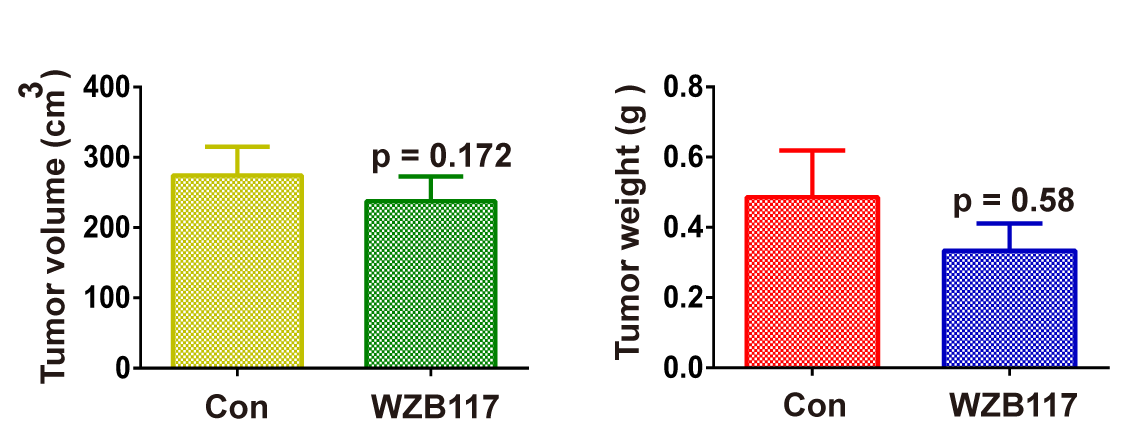

Supplement: Supplementary file 10 — Figure S9 [file 41389_2020_207_MOESM10_ESM.tif]
